# Moral distress among acute mental health nurses: A systematic review

**DOI:** 10.1177/09697330241238337

**Published:** 2024-03-15

**Authors:** Sara Lamoureux, Amy E Mitchell, Elizabeth M Forster

**Affiliations:** 60093 Gold Coast University Hospital; 5723 Griffith University; 1974 The University of Queensland; 5723 Griffith University; 5723 Griffith University

**Keywords:** Mental health/psychiatry, moral distress, nurse/nursing, acute inpatient, moral sensitivity

## Abstract

Moral distress has been identified as an occupational hazard for clinicians caring for vulnerable populations. The aim of this systematic review was (i) to summarize the literature reporting on prevalence of, and factors related to, moral distress among nurses within acute mental health settings, and (ii) to examine the efficacy of interventions designed to address moral distress among nurses within this clinical setting. A comprehensive literature search was conducted in October 2022 utilizing Nursing & Allied Health, Embase, CINAHL, PsychInfo, and PubMed databases to identify eligible studies published in English from January 2000 to October 2022. Ten studies met inclusion criteria. Four quantitative studies assessed moral distress among nurses in acute mental health settings and examined relationships between moral distress and other psychological and work-related variables. Six qualitative studies explored the phenomenon of moral distress as experienced by nurses working in acute mental health settings. The quantitative studies assessed moral distress using the Moral Distress Scale for Psychiatric Nurses (MDS-P) or the Work-Related Moral Stress Questionnaire. These studies identified relationships between moral distress and emotional exhaustion, depersonalization, cynicism, poorer job satisfaction, less sense of coherence, poorer moral climate, and less experience of moral support. Qualitative studies revealed factors associated with moral distress, including lack of action, poor conduct by colleagues, time pressures, professional, policy and legal implications, aggression, and patient safety. No interventions targeting moral distress among nurses in acute mental health settings were identified. Overall, this review identified that moral distress is prevalent among nurses working in acute mental health settings and is associated with poorer outcomes for nurses, patients, and organizations. Research is urgently needed to develop and test evidence-based interventions to address moral distress among mental health nurses and to evaluate individual and system-level intervention effects on nurses, clinical care, and patient outcomes.

## Introduction

The initial recognition of moral distress within nursing dates back to the 1970s and 1980s. During this time, medical schools were establishing bioethics courses and recognized the increasing interest from nurses and nursing students in this area. Nursing students’ concerns differed from those of medical students, however, in that they concentrated on feelings, practical situations, as well as nurse–patient and nurse–physician relationships.^1^ Andrew Jameton first defined moral distress in his 1984 text *Nursing Practice: The Ethical Issues* as ‘a challenge that arises when one has an ethical or moral judgement about care that differs from that of others in charge’.^2(p. 298)^ Although there have been several subsequent attempts to redefine moral distress, many have been poorly delineated. Additionally, there have been subsequent difficulties in distinguishing moral subjectivity from moral distress.^2^ With the increasing body of moral distress research, the ability to develop a clear definition of moral distress has complicated the attempts to study moral distress. Moreover, the rising number of cited causes, and effects, of moral distress has meant that its definition has expanded to now being an umbrella term that lacks precision, resulting in a wide-ranging phenomenon and causes. Without a coherent and consistent understanding of moral distress, its prevalence, effects and possible responses to moral distress are expected to be confusing and contradictory.^3^

Moral distress is a significant component of moral conflict which can negatively impact healthcare, including the success and quality of care, as well as workplace culture.^4^ Furthermore, institutions and professional colleagues can both impact the level of moral distress experienced by nurses and make it challenging, or impossible, for the nurse to act on their own best judgement.^5^ Moral distress can include the presence of unpleasant feelings or psychological conflict that arise when a clinician is aware of the ethically correct action to take in a given situation,^6^ but either internal or external factors restrain them from acting accordingly.^7^ Responses to the constraints experienced through moral distress include physical and emotional exhaustion combined with failure, frustration, anger, and guilt.^8^ Research has shown that moral distress ultimately diminishes nurses’ professional performance, negatively impacting patient care and nursing staff retention.^9^

Moral distress has been researched within a variety of clinical areas including intensive care, paediatrics, palliative care, and oncology settings.^9^ Giannetta et al.^10^ completed a systematic review centring on moral distress experienced by nurses within Intensive Care Units (ICUs). Findings from this review found a moderate level of moral distress among nurses within ICU settings. Causes of moral distress for ICU nurses included patient-level, unit/team-level, and system-level factors, and most studies in this review highlighted cost-control, end-of-life care, and futile care as causing the greatest moral distress among nurses.^10^

Eche et al.^11^ completed a systematic review focussing on moral distress among oncology nurses. Findings from this review identified that oncology nurses were likely to experience low to moderate frequency of moral distress, but a higher intensity of moral distress. Moral distress experienced by oncology nurses was a substantial predictor for burnout, end-of-life care decisions, provider communication, inability to deliver compassionate care, and work conditions.^11^

It has become recognized, internationally, that mental health nursing is a demanding professional speciality due to workplace stressors and challenges,^12^ including verbal and physical aggression,^13^ which can have a negative impact on the physical and psychological health, and the wellbeing of mental health nurses.^12^ These encounters not only negatively impact nurses and patients but also negatively impacts staffs’ ability to maintain a therapeutic environment.^13^

Acute mental health units are inpatient settings for individuals requiring involuntary, or voluntary, short-term treatment during the acute phase of a mental or psychiatric illness, depending on the severity of the patient’s symptoms, distress levels, and risk to themselves or to others.^7^ A significant component of the mental health nurses’ role within these environments is the therapeutic patient–nurse interpersonal relationships.^12^

Nurses may experience moral distress related to restrictions placed on patients,^14^ including involuntary admissions and restraints,^15^ working during staff shortages, observing unsafe patient care, inadequate clinical communication, economic constraints, increased clinical acuity, and the drive for efficiency.^16^ Moral distress has been identified as an occupational hazard for clinicians providing care to vulnerable populations, including care to mental health patients.^17^ However, little is known about the relationship between moral distress experienced by mental health nurses,^14^ how mental health nurses cope with repeated exposure to moral distress,^6^ and the relationship of moral distress with work-related phenomena, including burnout, job satisfaction, lack of empathy, and intention to resign from mental health units.^17^ The purpose of this review was to identify the prevalence of moral distress within acute mental health units, factors associated with moral distress experienced within these settings, and identifying the efficacy of any interventions currently in place. To address this, and other moral distress issues, the following questions were posed:

1. What is the prevalence, or level, of moral distress among mental health nurses in acute mental health settings?
2. What factors are associated with moral distress among mental health nurses in acute mental health settings?
3. What is the efficacy of interventions designed to address moral distress among nurses in acute mental health settings?

## Methods

The systematic review protocol was registered with PROSPERO (International Prospective Register of Systematic Reviews; CRD42022343038).

### Eligibility criteria

This systematic review considered primary research of all study designs and methodologies, including qualitative, quantitative, observational, experimental, and mixed methods research. The search was limited to peer-reviewed articles published between January 2000 and October 2022. January 2000 was selected as the earliest date to capture any quantitative studies which may have utilized Corley’s Moral Distress Scale and the Moral Distress Scale – Psychiatric Nurses within the acute mental health setting.

To be included, studies needed to meet the following inclusion/exclusion criteria:

1. *Types of studies.* Qualitative, quantitative, observational (prospective and retrospective cohort, case-control, and cross-sectional studies), experimental (randomized controlled trials, non-randomized controlled trials, and uncontrolled pre-post studies), and mixed methods studies were included in this review. Studies needed to be peer-reviewed and published in English to be considered.
2. *Types of participants.* Studies conducted with nurses working within acute inpatient mental health settings providing care to youth and/or adults were included. Studies that focused on non-nursing clinicians (physicians and allied health), clinical students, and nursing facilitators were excluded from this review, even if employed within an acute mental health setting. If studies reported on a mix of disciplines (e.g. a mixed sample of nurses and allied health), studies were only eligible for inclusion if separate data for nurses were reported.
3. *Types of settings.* Studies conducted in acute adolescent or adult inpatient mental health settings were eligible for inclusion. Studies conducted in acute child inpatient mental health units, mental health community programs, and non-acute inpatient mental health environments were excluded from this review. Additionally, non-mental health hospital units and community programs/teams were also not included in this review. Studies that were conducted in multiple clinical settings (e.g. acute care inpatient and outpatient/rehabilitation units) were included, so long as the data related to acute inpatient mental health settings could be extracted separately.

### Information sources

A systematic search of health-related databases was conducted to retrieve published articles via Nursing & Allied Health, Embase, CINAHL, PsychInfo, and PubMed. Database searches were conducted between October 8 and October 12, 2022. A manual search of Google Scholar was also completed to ensure that all relevant studies were captured.

### Search strategy

The search consisted of three overarching themes of moral distress, nursing, and acute psychiatric/mental health organization(s)/unit(s) with appropriate search terms and synonyms within each category to maximize search results. The research team collaborated with three expert librarians to determine the most appropriate search terms and refined the search strategy. Finalized search terms for title, abstract, and subject can be viewed in Supplementary Table S1.

### Selection process

Endnote was utilized to manage literature search sources as well as screening of titles and abstracts. Once duplicate articles were removed, title and abstract screening of the full search yield was undertaken by SL and AEM. Full texts of potentially eligible articles were retrieved and reviewed independently by SL and AEM who assessed each for eligibility. Where there was disagreement between the two reviewers regarding eligibility, a third reviewer (EMF) was consulted to reach a consensus.

### Data collection process

Data from each of the articles for inclusion in the review were extracted by SL using a form developed by the review team, and included author, year, country, study design, setting, methodology, methods, participant characteristics, intervention details (where relevant), and results.

### Data items

The primary outcomes were the prevalence or levels of moral distress and factors related to moral distress. Moral distress and factors related to moral distress could be assessed using any qualitative or quantitative assessment methods.

### Synthesis methods

A narrative synthesis approach was utilized for this review as it allows for the identification of the causes of particular social problems a particular community encounters and the factors that shape the implementation of interventions.^18^

## Results

### Study selection

**Figure 1.**
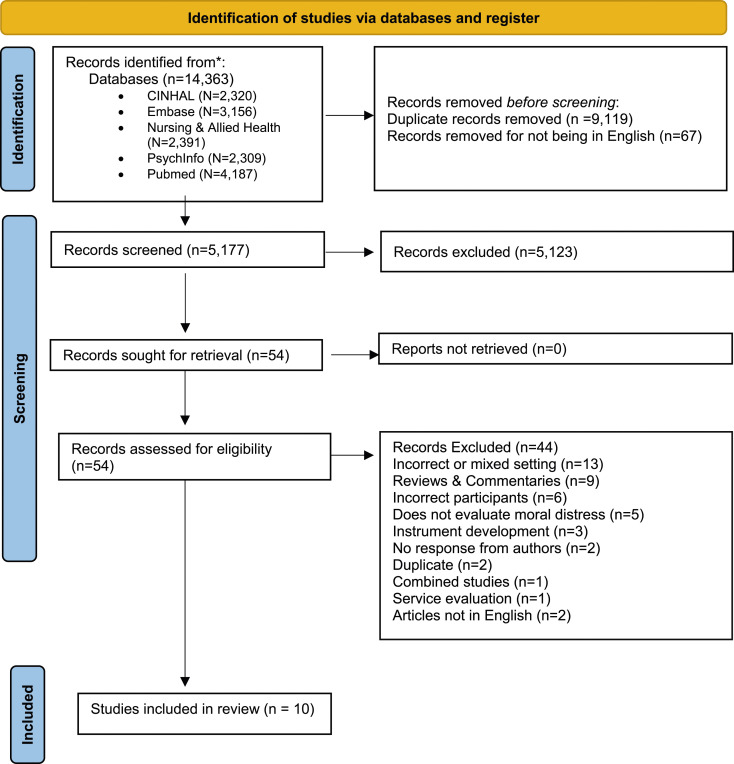
PRISMA flow chart for identification and screening of studies.

A total of 14,363 potentially eligible articles were identified through the search process. Of these articles, 9119 duplicates and 67 studies not published in English were removed. The remaining 5177 article titles and abstracts were screened, with 54 articles identified for full-text screening. SL emailed the authors of three studies to ascertain whether the research was conducted in an eligible setting (adolescent or adult acute inpatient mental health setting). All three studies were considered ineligible as the author of one study reported that the setting included both acute and sub-acute settings without distinguishing the results between the settings and the authors of the other two studies did not respond. Thus, a total of 10 articles met eligibility criteria and were included in the review (see Figure 1).

### Quality appraisal

Careful appraisal of the studies in any systematic review is a core component of systematic reviews and the Mixed Methods Appraisal Tool (MMAT) was used for quality appraisal.^19^ Critical assessment of the included quantitative, qualitative, and mixed methods studies was completed^19,20^ and five of the six qualitative studies met all five of the quality criteria with one qualitative study meeting four out of five quality criteria.^19,20^ All four of the quantitative studies met the quality criteria.

### Risk of bias in studies

Of the ten studies included, one study had one methodological quality criteria not met. Participants provided written answers to two questions as part of a questionnaire and answers were analysed as qualitative data. Seven themes were identified, with corresponding sub-themes.^21^ However, the identified themes in this research were not supported by evidence collected. The study met all other MMAT criteria and therefore was included in this review as it outlined moral distress as experienced by nurses within the acute inpatient setting.

### Study characteristics

Of the ten studies included, most were conducted in either Japan or Norway with three studies included from each country, respectively. The remaining four studies were from Canada, Ireland, Italy, and Sweden. Four of the studies were quantitative, while the remaining six studies were qualitative. Data extraction from the quantitative studies comprised of moral distress levels (including scores for frequency and intensity where reported), and its relationship with sense of coherence, job satisfaction, general health, participant demographics,^22^ and burnout.^8,9^ Hospital ethical climate, moral sensitivity, and work-related moral stress were also reported.^23^

Three of the quantitative studies utilized the Moral Distress Scale for Psychiatric Nurses (MDS-P),^8,9,22^ a self-report questionnaire used to measure moral distress within the acute mental health setting. These studies also included other self-reported questionnaire measures, such as the Sense of Coherence Scale,^22^ General Health Questionnaire,^22^ Job Satisfaction Scale,^22^ Maslach Burnout Inventory,^9^ and the Maslach Burnout Survey – General Survey,^8^ to assess the relationship between moral distress and other variables. Lutzen et al.^23^ did not utilize the MDS-P to assess moral distress. Instead, they utilized the Hospital Ethical Climate Survey,^23^ Moral Sensitivity Questionnaire^23^ and the Work-Related Moral Stress Questionnaire.^23^

#### Quantitative moral distress measures

The Moral Distress Scale was initially developed by Corley et al.^24^ in 2001 for use in the critical care settings^8^ and is based on Jameton’s definition of moral distress within nursing.^24^ The Moral Distress Scale for Psychiatric Nurses (MDS-P) was developed and validated by Ohnishi in 2010 and was based on the Moral Distress Scale initially developed by Corley and her colleagues.^8,22^ The MDS-P assesses for moral distress across three different subscales: unethical conduct by caregivers (6 items), low staffing (5 items), and acquiescence to patients’ rights violations (4 items).^8^ Each item uses a 7-point Likert response scale to measure either frequency or intensity of moral distress, and item scores are averaged to produce subscale and/or total scores.^8^ An average score of three on the MDS-P is the mid-point, with scores greater than three considered problematic.^22^

#### Other quantitative measures

In addition to using the MDS-P, Delfrate et al.^9^ assessed the relationship between moral distress and burnout using the Maslach Burnout Inventory (MBI). The MBI comprises of 22 items across three subscales: emotional exhaustion, depersonalization, and personal accomplishment, and higher scores indicate greater levels of burnout. Likewise, Ohnishi et al.^8^ utilized the Maslach Burnout Inventory – General Survey (MBI-GS).^8^ This scale has been used globally, and the Japanese version has been evaluated for reliability and validity.^8^ The MBI-GS consists of three subscales: exhaustion, cynicism, and professional efficacy.^8^ Lutzen et al.^23^ used the Moral Sensitivity Questionnaire (MSQ), Work-Related Moral Stress Questionnaire, and the Hospital Ethical Climate Survey to examine the relationship between work-related moral stress with moral climate and moral sensitivity within mental health settings.^23^ The 9-item Work-Related Moral Stress Questionnaire (WRMSQ) was developed specifically for Lutzen’s study to assess general symptoms related to stress experienced within the previous month.^23^ The 26-item Hospital Ethical Climate Survey was used to measure nurses’ experience of moral climate.^23^

#### Qualitative studies

Of the qualitative studies, one study utilized questionnaires which included two open-ended questions assessing how nurses responded when encountering an ethical issue and what resulted from their response to the ethical situation. The researchers then analysed the text answers using qualitative methods.^21^ Another study utilized a dialogue approach.^25^ The third study utilized semi-structured interviews,^26^ which consisted of open-ended questions alongside prompts to expand explanations.^26^

Two studies used in-depth interviews.^6,27^ These researchers used in-depth interviews to obtain insights into the participants subjective attitudes, experiences, motives, and thoughts.^6,27^ One study used one-to-one interviews, however, did not describe the type of interviews completed.^7^ Two of these studies also utilized focus groups.^6,7^

Data extraction from the qualitative studies focussed on factors associated with moral distress. Data extraction for all studies included sample size and settings. Data extraction for both quantitative and qualitative studies can be found in Table 1 and Table 2.

**Table 1.** Characteristics of quantitative studies.

Authors and country	Study aim	Study design	Sample setting	Measurement tool(s)	Level of moral distress and correlates
Ando and Kawano, 2018 (Japan)	(1) Examine relationships between moral distress, sense of coherence, mental health, and job satisfaction (2) Identify the strongest predictor of job satisfaction	Cross-sectional study	130 psychiatric nurses from a large national hospital	Moral distress: Moral distress scale for psychiatric nurses (MDS-P) Other: Sense of Coherence Scale (SOC-12), Job Satisfaction Scale, and General Health Questionnaire (GHQ-12)	MDS-P intensity total score *M* = 2.11 (*SD* = 1.45). MDS-P subscale scores: • Unethical conduct by caregivers *M* = 2.01 (*SD* = 1.37) • Low staffing *M* = 1.69 (*SD* = 1.39) • Acquiescence to patients’ rights violations *M* = 2.64 (*SD* = 1.58) Negative correlations between moral distress (MDS-P) subscale scores and sense of coherence subscale scores (comprehensibility, manageability, and meaning) *r* = −0.17 to −0.31, *p* < .05). Negative correlations between moral distress (MDS-P) subscale scores and job satisfaction (*r* = −.30 to −.46, *p* < .05)
Delfrate, Ferrara, Spotti, Terzoni, Lamiani, Canciani, and Bonetti, 2018 (Italy)	To assess the presence of moral distress among mental health nurses and test for relationship between moral distress and burnout	Cross-sectional study	228 psychiatric nurses from Psychiatric units across four public hospitals: 7 inpatient units, 7 outpatient clinics, and 9 rehabilitation centres	Moral distress: Moral Distress Scale for Psychiatric Nurses (MDS-P) Other: Maslach Burnout Inventory (MBI)	Higher moral distress scores for nurses in inpatient settings compared to outpatient units or rehabilitation centres (*p* = .006). MDS-P intensity total score median = 2 (no range reported) Positive correlations between moral distress and two out of three MBI subscales (emotional exhaustion, r = 0.28, *p* < .0001; depersonalization, r = 0.20, *p* < .0001). No correlation between moral distress and personal accomplishment
Ohnishi, Ohgushi, Nakano, Fujii, Tanaka, Kitaoka, Nakahara, and Narita, 2010 (Japan)	To (1) develop and appraise the Moral Distress Scale for Psychiatric Nurses (MDS-P); (2) use the MDS-P to examine moral distress experienced by Japanese psychiatric nurses; and (3) examine the relationship between moral distress and burnout	Cross-sectional study	264 psychiatric nurses from 1 national university hospital, 1 public hospital, and 4 private hospitals	Moral distress: Moral Distress Scale for Psychiatric Nurses (MDS-P) Other: Maslach Burnout Inventory – General Survey (MBI-GS)	MDS-P intensity total score *M* = 2.86 (*SD* = 1.79) MDS-P frequency total score *M* = 2.47 (SD = 1.71) Positive correlations between moral distress intensity and MBI-GS subscales (cynicism, *r* = 0.30; exhaustion, *r* = 0.23). Positive correlations between moral distress frequency and MBI-GS subscales (cynicism, *r* = .42; exhaustion, *r* = 0.31). Only weak correlations between moral distress intensity or frequency and demographic characteristics (age and years of nursing/psychiatric nursing experience; rho<0.20). No differences in moral distress intensity or frequency total scores or subscale scores between men and women or registered and licenced practical nurses. Significant differences between hospitals for moral distress subscale low staffing (frequency total score) only
Lutzen, Blom, and Ewalds-Kvist, 2010 (Sweden)	To examine the relationship between work-related moral stress, moral climate, and moral sensitivity within mental health nursing	Cross-sectional study	49 psychiatric nurses from 4 acute psychiatric hospital wards within the same psychiatric clinic in central Sweden	Moral distress: Work-Related Moral Stress Questionnaire Others: Hospital Ethical Climate Survey and Moral Sensitivity Questionnaire	Negative correlations between moral stress and both moral climate (*r* = −.398, *p* = .004) and experience of moral support (*r* = −.397, *p* = .005). Positive correlation between moral stress and experienced moral understanding (i.e. moral awareness; *r* = .414, *p* = .002).

**Table 2.** Characteristics of qualitative studies.

Authors, country	Study design	Study aim	Sample setting	Factors associated with moral distress
Ando and Kawano, 2016 (Japan)	Mixed methods	(1) Examine the relationship between moral distress, sense of coherence, mental health, and job satisfaction (2) Clarify the most predictive variable to job satisfaction	136 nurses acute mental health unit	Seven key areas causing moral distress: • Lack of action and no change • Experiencing problems and feeling gloomy • Pointing out misconducting and being hurt • Consulting among staff and resolution or lack thereof • Consulting with a physician and getting positive or negative response • Searching for and providing evidence-based care • Thinking for themselves
Austin, Bergum, and Goldberg, 2003 (Canada)	Hermeneutic phenomenological approach	The objectives of the research are threefold: (1) to identify care situations which mental health practitioners find morally distressing; (2) to describe practitioners experience in raising and resolving ethical concerns regarding patient care; and (3) to identify environmental supports and obstacles which influence reflective, ethical practice	6–9 nurses acute mental health unit	Moral distress experienced due to: • Lack of time • Moving quickly from one patient to another • Feeling pressured to act quickly • Absence of respect ‘which is essential to ethical practice’
Deady and McCarthy, 2010 (Ireland)	Descriptive	The aim of the study was to explore psychiatric nurses’ experiences of moral distress within acute care settings	8 nurses acute mental health unit	External sources of moral distress • Professional and legal conflict • Professional autonomy and scope of practice • Standards of care and client autonomy • Increased policy and legal frameworks to protect patient rights as further sources of additional ethical challenges for practitioners .
Jansen, Danbolt, Hanssen, Hem, 2022 (Norway)	Interviews pre- and post-program implementation	To investigate whether diffuse Political and therapeutic ideals together with new legislations may present nurses working within acute psychiatric care with conflicting interests and challenges that may cause moral distress	30 nurses Acute mental health units across two mental health hospitals 16 participated in in-depth interviews and 14 participated in focus groups	Moral distress experienced due to: • The inability to safeguard other patients • Challenging behaviours and risk of violence • Use of restraints and coercion by staff • Criticisms, and descriptions of psychiatric units from the media • Feelings of isolation from society, family, and friends who may be unable to appreciate the work, intense experiences, dilemmas, and fears on an acute psychiatric unit • Unit architecture and size also a hindrance for fewer restraints and coercion
Jansen, Hem, Danbolt, and Hanssen, 2022 (Norway)	Qualitative: In-depth interviews and focus groups	To investigate how nurses attempt to cope with moral distress	30 nurses Acute mental health units across two mental health hospitals 16 participated in in-depth interviews and 14 participated in focus groups	Moral distress experienced due to: • Worry related to quality of treatment and care provided • Lack of resources • Some reported difficulty in not taking work home with them • Feelings of frailty, emptiness, tiredness, and grumpiness when getting home • Lacking strength and initiative • Worry regarding becoming emotionally detached from work • Exposure to workplace violence • Participation in coercive treatment or treatment they disagreed with
Jansen, Hem, Danbolt, and Hanssen, 2020 (Norway)	Individual in-depth interviews	To determine the source of moral distress and what characterizes moral distress in acute mental care nursing settings	16 nurses 12/16 psychiatric specialists acute mental health unit	Moral distress experienced due to: • Nurses’ perceptions of patient needs and their own capacity to provide care • Exposure to violence and coercion • Feelings of frustration, mentally tired, anger, inadequacy, sadness, and loss • Doubt, loyalty, and moral distress

### Prevalence of moral distress

#### Quantitative studies

Ando and Kawano focussed their research on the intensity of moral distress among mental health nurses in mental health units.^22^ They reported an overall mean score of moral distress intensity on the MDS-P of 2.11 (SD = 1.45).^22^ The highest subscale score was for moral distress due to acquiescence to patients’ rights violations (*M* = 2.64, *SD* = 1.58), followed by unethical conduct by caregivers (*M* = 2.01, *SD* = 1.37) and low staffing (M = 1.69, *SD* = 1.39).^22^ These researchers found negative correlations between the moral distress subscales and both sense of coherence and job satisfaction, with greater moral distress associated with poorer sense of coherence and worse job satisfaction.^22^

Delfrate et al.’s^9^ study likewise utilized the MDS-P to assess moral distress of nurses working within mental health inpatient, outpatient, and rehabilitation settings in Italy. The median overall MDS-P score for mental health inpatient nurses was 2, which was higher compared to the median scores for mental health nurses working in outpatient units and rehabilitation centres.^9^ They also utilized the Maslach Burnout Inventory (MBI) to assess the relationship between moral distress and burnout, finding positive correlations between moral distress and the emotional exhaustion, and depersonalization (but not personal accomplishment) subscales of the MBI.^9^

Ohnishi et al.^8^ utilized the MDS-P to measure both the intensity and frequency of moral distress among nurses in Japan. Mean scores for overall intensity and frequency of moral distress were 2.86 (SD = 1.79) and 2.47 (SD = 1.71), respectively.^8^ There were moderate positive correlations between both the intensity and frequency of moral distress and the exhaustion and cynicism subscales within the MBI-GS.^8^ There were only weak correlations between moral distress intensity and frequency and demographic characteristics, and no differences in moral distress intensity or frequency total scores or subscale scores between men and women or registered and licenced practical nurses. Finally, there were significant differences between different hospitals for moral distress scores for the low staffing subscale (frequency total score) only.^8^

Finally, Lutzen et al.^23^ found negative correlations between moral stress and both moral climate and experience of moral support. However, there was a positive correlation between moral stress and experienced moral understanding.^23^

#### Qualitative studies

Thematic analysis was utilized to identify themes within the qualitative data. Braun and Clarke’s inductive approach was used to identify themes based on the research presented.^28^ Within the six qualitative studies, three themes influencing the presence of moral distress were found. These included aggression and coercion, insufficient resources, as well as legal conflict and professional approaches. An additional theme highlighted within the qualitative studies focused on nurses’ coping mechanisms.

#### Aggression and coercion

There has been an increase in patients experiencing mental illness due to synthetic drug use.^7^ This combination of substance use with fewer inpatient mental health beds has resulted in patients that are admitted who are considerably more unwell as compared to patients admitted to acute mental health inpatient units ten years ago.^7^ Nurses reported experiencing serious verbal^7^ and physical^27^ aggression from patients, as well as observing physical aggression from patients towards inanimate objects and other patients,^7^ thus creating an apprehensive atmosphere.^7^ There are several factors that impact nurses’ ability to utilize restraints, including low predictability of aggression, architecture and size of the unit, limited documentation on when to use restraints, and the pressure to reduce the use of restraints.^7^ Nurses reported that they wanted to use the least restrictive processes, ensuring that all other avenues had been utilized first^7^ and also felt the need to increasingly tolerate violence and to only utilize restraints when violence was imminent.^7^ Nurses reported feeling shame and guilt in relation to not preventing patient violence or aggressive behaviour,^6^ safeguarding other patients from this aggression^7^ and believe that they could have prevented injury to others if they had restrained the patient earlier, leading to moral distress as a result.^7^

The current treatment philosophy to allow patients to de-escalate on their own also creates an ethical dilemma for nurses as this process can negatively impact other patients and creates doubt among staff.^27^ Nurses expressed frustration regarding the minimal attention to patient violence and how this impacts nurses.^27^ Nurses have also identified that using restraints often leaves them experiencing unpleasant feelings.^7^ Repeated exposure to aggression places nurses at risk for vicarious trauma, PTSD symptoms, shame, self-blame, occupational stress, guilt, skeletal problems, and burnout syndrome.^7^

The use of coercion in situations that could have otherwise been resolved through improved staffing or the unit’s routines had the nurses feeling uncomfortable,^27^ guilty, and shameful.^6^ Nurses’ frustrations were also experienced when a patient’s autonomy was limited due to their disruptive behaviour being caused by a colleague’s communication style or personality.^27^

#### Insufficient resources

Lack of time has been found to negatively impact patient care.^27^ Nurses reported that time was considered essential in providing necessary care, including building relationships, engaging in therapeutic conversations, patient motivation and reassurance, supervising patients in seclusion, and engaging in clinical assessments.^27^ Additionally, insufficient time inhibited nurses from actually knowing who their patients were beyond their diagnosis and medications^25^ and increased nurses’ doubting the quality of their assessments.^27^

Insufficient competency and time can lead to superficial treatment, including reduced follow-up for patients experiencing suicidal thoughts, potentially reducing time with patients at risk of self-harm and suicide – both of which require immense time from nurses.^27^ Furthermore, lack of time may lead to more disrupting behaviours and use of coercion.^27^ As a result of the limited time spent with patients, participants reported feelings of disgust^25^ and increased worry, vulnerability, and guilt in relation to not being able to spend the necessary time with patients at risk of suicide or self-harm.^27^

Nurses reported the lack of resources frustrating and often led to patients not receiving the care they required.^6^ Nurses reported experiencing inexcusable behaviour by both unskilled and professional staff, increasing the risk of dangerous situations.^6^ Nurses reported experiencing challenges when delegating suicidal patients, patients requiring seclusion, or patients at risk of increased violence to untrained clinicians as these clinicians may not comprehend the seriousness, or have the same sense of responsibility, as permanent clinicians.^6^ The use of untrained clinicians increased nurses’ worry as they spent more time monitoring these clinicians than they spent time with patients.^6^ When nurses raised concerns related to staffing issues and impact on patient safety, they were invalidated, and they reported they did not see any changes.^6^ Additionally, the lack of time,^25^ coupled with inadequate staffing,^6^ may have led to clinicians viewing patients as being problems rather than individuals.^25^

#### Legal conflict and professional approach

Deady and McCarthy’s research found that patient care failing to meet professional and personal criteria for best practice may lead to moral distress.^26^ This research found that, overall, nurses did not feel moral distress if coercion and seclusion practices were prescribed by physicians, legal, and applied appropriately. However, the participants in Deady and McCarthy’s research did report that they experienced moral distress when coercion and seclusions were applied due to medical interventions being insufficient, delays in being prescribed, or prescribed inappropriately.^26^

Changes in therapeutic, political, and legal ideals, particularly around the treatment of mental health patients have made it more challenging to admit a patient to a psychiatric unit with an involuntary status, increasing the number of voluntary admissions, and the length of these admissions due to the necessity for longer assessment periods prior to changing the patient’s voluntary inpatient status to an involuntary admission status.^7^ This results in nurses reporting feelings of sadness and frustration as they witnessed the deterioration of patients’ health as these patients declined care, and had to wait for days before they received adequate treatment and medications.^7^ As these voluntary patients became increasingly unwell, families did not want the patient to be discharged; however, voluntary patients cannot be kept on the unit against their will.^6^ Nurses’ reported feeling personally responsible for motivating patients to remain in hospital and to accept treatment, and tended to utilize pressure or persuasion rather than motivation to achieve this.^7^

Austin et al.’s^25^ research found that nurses’ experiences of moral distress were often situated around the responsibilities and expectations society had placed on healthcare practitioners. Being given such responsibilities were often connected with being considered trustworthy; however, when nurses were given such responsibilities, they were not always provided with the necessary control and power.^25^

Nurses perceived other members of the interdisciplinary team, such as physicians and allied health clinicians, to have greater influence of clinical decision-making, either due to their status within the professions or by mental health law.^26^ Additionally, other disciplines involved in patient care were able to move freely and leave the unit, whereas nurses were left to spend more time with patients and observed the ongoing deterioration of patient health.^26^ This became more prominent when medical care was delayed, and conflicts between patients increased.^26^ Nurses also reported that their assessments, particularly around patient deterioration, were not always heard by other disciplines.^26^ Nurses also reported frustration with the limited post-incident discussion, leaving nurses feeling that incidents and their moral concerns remained unresolved.^26^

Despite nurses’ not wanting to tolerate illegal practices, participants reported they felt the need to get along with their colleagues despite sometimes viewing their colleagues’ practice as poor.^26^ Participants within Deady and McCarthy’s research reported that they found it arduous to challenge colleagues, including by questioning the nurse’s competence or not providing support at key clinical patient cares, as it may have resulted in being isolated from the team, even if being isolated subtly.^26^ Acute mental health nurses have reported that it can be distressing to challenge a peer’s standard of practice^26^ and reported experiencing negative outcomes such as feeling hurt, leading to helplessness if they did so.^21^

Finally, participants in Deady and McCarthy’s research reported that restricting patients’ autonomy increased nurses’ moral distress.^26^ The example highlighted in this research included a palliative patient’s discharge from hospital being prevented as the patient expressed interest in seeking euthanasia overseas, and this was seen as an articulation of suicidal intent. The nurse reported feeling that the patient was being unfairly heard and that the patient should be able to make their own end-of-life decisions.^26^

#### Coping with moral distress

The build-up of moral distress can lead to moral residue, which can, in turn, lead to long-term negative feelings and a decrease in quality patient care. As a result, improving coping abilities is necessary.^22^ Jansen et al.^6^ highlighted that coping mechanisms consisted of not taking work home and speaking up versus loyalty.^6^

#### Efficacy of interventions for moral distress

Exploring coping processes can lead to better understanding of the kinds of skills, supports, and structures nurses require to identify strategies to mitigate moral distress.^6^ Of the articles included in this review, none explicitly assessed the efficacy of interventions, or considered possible interventions, as part of their research. Although not overtly explored in the available research, some research has reported that mentoring groups could be helpful to manage moral distress and that the process of ‘sorting work’ was essential.^6^ It was also noted that unit culture impacts how nurses express their concerns and have their experiences acknowledged.^6^

## Discussion

This mixed methods systematic review examined the prevalence, factors associated with, and the efficacy of interventions for addressing moral distress among mental health nurses working in acute mental health settings. Nurses’ experience of moral distress varies by setting.^15^ Nurses within acute mental health inpatient settings have been shown to experience greater moral distress than their counterparts in outpatient units and rehabilitation centres which may be due to working within closed environments, with restraint procedures more frequently utilized than these other settings.^9^

Moral distress within mental health settings has been identified as an occupational hazard for professionals not only because they are caring for vulnerable communities^17^ but also due to mental health patients experiencing mental illness human rights violations^16^ and restrictions on patients’ freedom, including involuntary hospitalizations.^15^

Ohnishi et al.’s^8^ and Delfrate et al.’s^9^ research found positive correlations between moral distress and burnout.^8,9^ Burnout may be an unintended consequence of not detecting moral distress early^15^ and can become a serious problem if failed to be noticed by management.^29^ For example, burnout can increase hospital errors which in turn significantly impacts quality of care, safety culture, and the resilience of care providers^29^ and can lead to low job satisfaction and poor nursing retention.^17^

For the nurse, the physical and psychological effects of moral distress can be long lasting^8^ and harmful to their professional and personal wellbeing.^16^ Moral distress can contribute to medication errors, poor communication between clinicians, and negative work outlook.^17^ Further unresolved moral distress can lead to moral residue, which results in the build-up of moral distress through subsequent experiences, making it problematic to fully resolve the initial moral distress,^30^ leading to negative feelings of guilt, sadness, powerlessness, shame, despondency, angst, resignation,^7^ desensitization and disengagement of clinicians,^30^ and negative impacts on nurse retention^5^ as nurses leave their work environment and nursing altogether.^8^

The quantitative studies included in this review showed similar results in the level of moral distress among inpatient mental health nurses. Moral distress among acute mental health nurses was found to be low,^22^ moderately low,^8^ and medium-low.^9^ In comparison to other clinical areas, oncology nurses experience low to moderate levels of moral distress, with some variations in intensity and frequency. However, evidence has suggested that critical care nurses experience higher levels of moral distress as compared to nurses in other clinical settings, including mental health.^11^ Critical care nurses may experience quantitatively higher moral distress levels due to the complexities of caring for patients with critical needs, which is often associated with the burden of uncertainty and difficulties in treatment-related decision-making.^31^

The severity and frequency of moral distress is associated with an assortment of factors.^32^ The studies in this review found that the external factors outnumbered the internal factors. These external factors included aggression and violence.^7,27^ insufficient resources,^6,7,21,25–27^ legal conflicts and professional approaches,^6,7,26^ as well as loyalty^6,7^ and support.^6^ Moral distress has been associated with diverse cognitive and emotional consequences and can lead to inattention or distraction.^33^

Given the pervasiveness of moral distress and its potentially negative impact, organizations and clinicians need to intervene.^34^ However, there have been few evidence-based interventions that have been found to alleviate the negative effects of moral distress,^35^ and none have been tested with nurses in psychiatric settings.

The systematic reviews that have reviewed moral distress interventions have identified that there is limited evidence of interventions currently in place.^34^ Moral distress interventions that have been implemented, have been primarily implemented through educational and reflective workshops.^34^ Interventions to improve nurses’ coping strategies related to moral distress include assertiveness training and education aimed to enhance critical clinical approaches.^17^ Assertiveness training will allow for nurses to overcome apparent hierarchy related challenges with their nursing and interdisciplinary colleagues. Additionally, education and professional development is likely to increase the accountability of early career nurses and other members of the multidisciplinary team.^17^ Further approaches for moral distress interventions require a more standardized approach, utilizing validated tools, and consistent follow-up for these interventions to be considered effective. These interventions need to be available across the hospital setting and not be unit specific given the pervasiveness of moral distress across the hospital system.^34^

## Limitations

The inclusion of a small number of studies published in English and the limited number of studies within the acute inpatient mental health setting may limit the generalizability of these findings. Additionally, the studies included in this review were from a limited number of countries. As such, cultural and geographical influences which may impact how moral distress is experienced in these countries may not be generalized globally.

## Conclusion and recommended future research

Nurses within acute mental health settings experience various factors that can lead to moral distress. These include insufficient resources, inadequate and/or inappropriate staffing, relationships with other disciplines, exposure to aggression and violence, participation in coercive care, and experiencing negative feelings outside the workplace because of their work.

There are two areas of note that need further research regarding moral distress. First, there are few studies that focus on interventions for moral distress for nurses who provide direct patient care. Further research is necessary to develop and test evidence-based interventions to address moral distress among nurses and to evaluate the effect this has on nurse, patient and organizational outcomes.^36^

The second area that may require additional research is how moral distress affects men versus women. The only study to assess this was Lutzen et al.^23^ Jensen et al.^27^ did not assess the impact of moral distress on men and women but acknowledged that, given the low participation rate of men in their study, more research will be needed to examine gender differences in moral distress with samples in which more male nurses are represented.^27^ There is a scarcity of research on how gender influences the experience of moral distress for which this relationship can be evaluated and compared. Furthermore, gender bias within nursing research has been well-documented.^37^

Nurses working in acute mental health settings experience levels of moral distress that are impacted by aggression, limited resources, and legal and professional obligations.^38^ Mental health nurses are the largest group of clinicians within mental health settings, yet many new mental health nurses are more likely to leave their jobs within the initial two years within mental health to pursue other opportunities to develop their clinical abilities and skills.^39^ The combination of increasing patient acuity^38^ with an expected shortage of mental health nurses in many countries including Australia by 2030^12^ highlights the necessity to engage in moral distress research and related interventions for mental health nurses who may experience moral distress.

## Supplemental Material

Supplemental Material - Moral distress among acute mental health nurses: A systematic review Supplemental Material for Moral distress among acute mental health nurses: A systematic review by Sara Lamoureux, Amy E Mitchell, and Elizabeth M Forster in Nursing Ethics.
