# Supplementary material for: Moral distress among acute mental health nurses: A systematic review
Source: Nurs Ethics. 2024 Mar 15;31(7):1178–95. doi: 10.1177/09697330241238337 (PMC11492570; doi:10.1177/09697330241238337)
Supplement: Supplemental Material - Moral distress among acute mental health nurses: A systematic review [file sj-pdf-1-nej-10.1177_09697330241238337.pdf]

Supplementary Table S1
Nursing & Allied Health (8 Oct 22)

**Moral Distress**		
S1	ti(moral* OR stress OR "moral distress" OR integrity OR ethic* OR cop*) OR ab(moral* OR stress OR "moral distress" OR integrity OR ethic* OR cop*)	277,590
S2	MESH.EXACT.EXPLODE("Social Responsibility:F.01.829.500.760") OR MESH.EXACT.EXPLODE("Social Responsibility:K.01.752.566.869")	2,116
S3	(MESH.EXACT.EXPLODE("Nursing Staff:N.02.360.680") OR MESH.EXACT.EXPLODE("Nursing Staff:M.01.526.485.680")) AND MESH.EXACT.EXPLODE("Ethics:N.05.350")	277
S4	ti(organizational) AND MESH.EXACT.EXPLODE("Ethics:N.05.350")	35
S5	ab(organizational) AND MESH.EXACT.EXPLODE("Ethics:N.05.350")	144
S6	MESH.EXACT.EXPLODE("Stress, Physiological") AND ti(psychological)	14
S7	MESH.EXACT.EXPLODE("Stress, Physiological") AND ab(psychological)	69
S8	ti(moral*) OR ab(moral*)	15,423
**S9**	**S1 OR S2 OR S3 OR S4 OR S5 OR S6 OR S7 OR S8**	**279,296**
**Nursing**		
S10	ti(("mental health" OR psychiatr*) near/4 (nurs* or clinician*)) OR ab(("mental health" OR psychiatr*) near/4 (nurs* or clinician*))	10,128
S11	ti(nurs*) OR ab(nurs*) AND MESH.EXACT.EXPLODE("Education, Nursing") AND ti(level) OR ab(level)	717,909
S12	MESH.EXACT.EXPLODE("Nursing Staff:N.02.360.680") OR MESH.EXACT.EXPLODE("Nursing Staff, Hospital:M.01.526.485.740.523") OR MESH.EXACT.EXPLODE("Nursing Staff:M.01.526.485.680") OR MESH.EXACT.EXPLODE("Nursing Staff, Hospital:N.02.360.740.523") OR MESH.EXACT.EXPLODE("Nursing Staff, Hospital:M.01.526.485.680.490") OR MESH.EXACT.EXPLODE("Nursing Staff, Hospital:N.02.360.680.490")	8,106
S13	(MESH.EXACT.EXPLODE("Psychiatric Nursing:N.02.421.533.778") OR MESH.EXACT.EXPLODE("Psychiatric Nursing:H.02.478.676.710"))	1,448
**S14**	**S10 OR S11 OR S12**	727,614
**Organization/Unit**		
S15	ti(("mental health" OR psychiatr*) near/3 (ward OR unit OR inpatient OR "in patient" OR setting OR hospital OR nurs*)) OR ab(("mental health" OR psychiatr*) near/3 (ward OR unit OR inpatient OR "in patient" OR setting OR hospital OR nurs*))	17,623
S16	ti(acute near/2 ("mental health" or psychiatr*)) OR ab(acute near/2 ("mental health" or psychiatr*))	1,430
S17	MESH.EXACT.EXPLODE("Hospitals") AND ti(psychiatric) OR ab(psychiatric)	37,848
S18	MESH.EXACT.EXPLODE("Hospitals") AND ab(psychiatric) OR ti(psychiatric)	12,641
**S19**	(MESH.EXACT.EXPLODE("Psychiatric Department, Hospital:N.02.278.216.500.968.641") OR MESH.EXACT.EXPLODE("Psychiatric Department, Hospital:N.04.452.442.452.422.641")) OR MESH.EXACT.EXPLODE("Hospitals, Psychiatric")	**1,115**
**S20**	**S15 OR S16 OR S17 OR S18 OR S20**	**51,063**
**S21**	**S9 AND S14 AND S20**	**2,391**
